# KLK5 and KLK7 drive cervical carcinoma via KLK14-dependent RhoA and NF-κB pathways

**DOI:** 10.1016/j.tranon.2025.102488

**Published:** 2025-08-05

**Authors:** Gabriel Viliod Vieira, Rodrigo Alberto Alves da Silva, Letícia Andrade Costa, João Paulo Bianchi Ximenez, Margarita Lamprou, Mateus Gonçalves Miranda, Vitor de Moura Arrais, Bruna Miyoko Ikenaga de Brito, Elaine Zayas Marcelino da Silva, Bruno Belmonte Martinelli Gomes, Carol Kobori da Fonseca, Márcia Gaião Alves, Camila Aparecida Coelho Brazão, Kevin Luiz Lopes-Delphino, Laura Miguel Rodríguez, Thiago Mattar Cunha, Ana Paula Lepique, Constantinos M. Mikelis, Raphael Sanches Peres, Wilson Araújo Silva, Leandro Machado Colli, Silvana Maria Quintana, Katiuchia Uzzun Sales

**Affiliations:** a Department of Cell and Molecular Biology and Pathogenic Bioagents, Ribeirao Preto Medical School, University of Sao Paulo, Sao Paulo, Brazil; b Department of Medical Imaging, Hematology, and Clinical Oncology, Ribeirao Preto Medical School, University of Sao Paulo, São Paulo, Brazil; c Department of Clinical Analyses, Toxicology and Food Science, School of Pharmaceutical Sciences of Ribeirao Preto, University of São Paulo, Sao Paulo, Brazil; d Department of Pharmacy, University of Patras, Patras, Greece; e Department of Gynecology and Obstetrics, Ribeirao Preto Medical School, University of Sao Paulo, Sao Paulo, Brazil; f Department of Pharmacology, Ribeirao Preto Medical School, University of Sao Paulo, Sao Paulo, Brazil; g Department of Immunology, Biomedical Sciences Institute, University of Sao Paulo, Sao Paulo, Brazil; h Department of Genetics, Ribeirao Preto Medical School, University of Sao Paulo, Sao Paulo, Brazil

**Keywords:** Cervical carcinoma, High-grade squamous intraepithelial lesion, Kallikrein 5, Kallikrein 7, Kallikrein 14, RhoA and NFkB, Serine proteases

## Abstract

**Background:**

The global prevalence of Human Papillomavirus (HPV) infection is approximately 12%, which significantly contributes to the development of cervical cancer as HPV is a key driver of tumorigenesis. However, the precise mechanisms by which HPV promotes carcinogenesis and the involvement of additional components in this process remain poorly understood.

**Objective:**

Given the evidence supporting the critical role of serine proteases in carcinogenesis, we investigated their contribution to cervical cancer development in the context of HPV-mediated carcinogenesis.

**Methods:**

Human biopsies were analyzed to assess the expression of serine proteases, including matriptase, kallikrein 5 (KLK5), and kallikrein 7 (KLK7), as well as their endogenous inhibitors. Mechanistic studies were conducted using genetically engineered mice, bulk RNA-seq, and reporter assays to elucidate the role of these proteases in HPV-dependent cervical carcinogenesis.

**Results:**

Our findings demonstrate increased expression of matriptase, KLK5, and KLK7 in early cervical carcinogenesis. Furthermore, the absence of both KLK5 and KLK7 ameliorates the HPV-dependent phenotype via modulation of KLK14 activation. KLK14 exhibits a pro-tumorigenic effect by regulating PAR-2-dependent RhoA and NF-κB signaling pathways.

**Conclusion:**

This study underscores the critical roles of serine proteases KLK5, KLK7, and KLK14 in cervical carcinogenesis, suggesting that these serine proteases are promising targets for the development of novel therapeutic strategies in cervical cancer**.**

## Highlights

• Serine proteases KLK5, KLK7, and KLK14 are upregulated in HPV-driven cervical cancer.
• KLK5 and KLK7 deletion mitigates HPV-induced tumorigenesis via KLK14 modulation.
• KLK14 promotes tumorigenesis by activating PAR-2/RhoA/NF-κB signaling pathways.
• Targeting serine proteases may offer novel therapeutic strategies for cervical cancer.

## Graphical abstract

**Figure:**
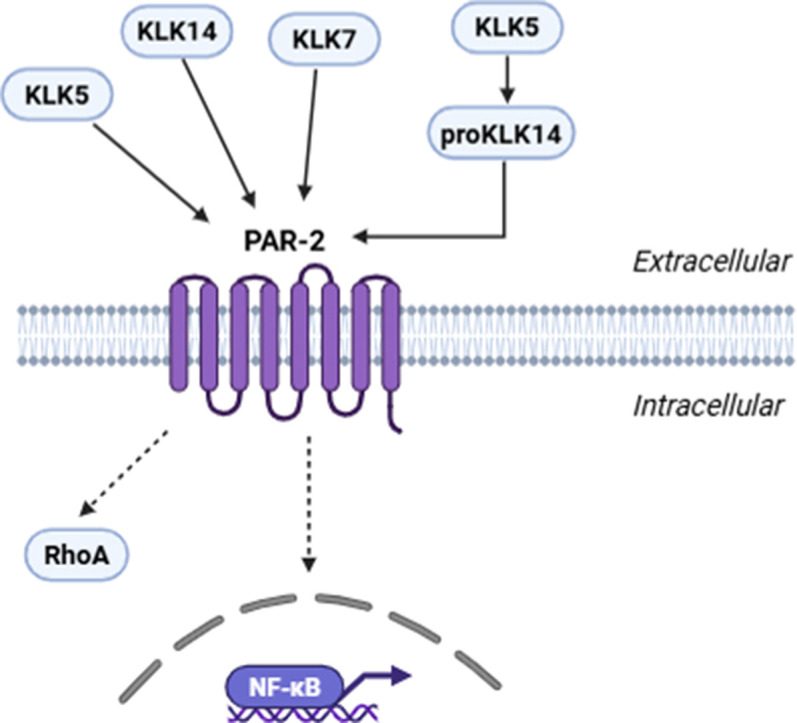

## Introduction

Cervical cancer is the third leading cause of death among women, representing a significant public health challenge, particularly in low- and middle-income countries [1]. While the mechanisms underlying cervical cancer development are not fully understood, Human Papillomavirus (HPV) is recognized as a key driver [2,3]. A critical event in tumor progression is the integration of the HPV genome into the DNA of basal epithelial cells. These mitotically active cells can transform from normal mucosa to invasive carcinoma [4]. From more than 120 identified HPV subtypes, HPV 16 and 18 are the most strongly associated with malignant lesions, especially cervical cancer [5].

Abnormal expression of serine proteases also contributes to tumor development by facilitating pathological processes such as tumor initiation, progression, invasion, and metastasis [6]. The imbalance of several serine proteases’ expression, including matriptase and kallikreins, has been linked to various human cancers [[7], [8], [9], [10], [11], [12]]. Moreover, dysregulation of matriptase's cognate inhibitors, such as hepatocyte growth factor activator inhibitor type 1 (HAI-1) and type 2 (HAI-2), has been observed in carcinomas [9,[11], [12], [13], [14]]. Notably, reduced HAI-1 expression levels are strongly associated with poor cervical carcinoma prognosis [9]. Additionally, the serine proteases kallikrein 5 (KLK5) and kallikrein 7 (KLK7) are overexpressed in malignant cells [[10], [11], [12],[15], [16], [17], [18], [19]]. Interestingly, the kallikreins' cognate inhibitor lymphoepithelial Kazal-type-related inhibitor (LEKTI) inhibits matriptase-dependent carcinogenesis by regulating kallikreins' function [15].

While some studies have linked the dysregulation of serine protease expression to carcinogenesis, their role in tumor development in cervical cancer remains poorly understood. Herein, we demonstrated an aberrant expression of matriptase, KLK5, and KLK7 in human biopsies from patients with early cervical cancer (high-grade intraepithelial lesion - HSIL). Using genetically engineered mice, we observed that the double deficiency of *Klk5* and *Klk7* restored the phenotype of mice bearing HPV-dependent tumors. Bulk RNA-seq analysis from lesions of transgenic mice expressing oncogenes related to HPV infection revealed that *Klk5* and *Klk7* regulate *Klk14* expression, which activates RhoA and NF-κB signaling pathways, thereby promoting tumor progression. Collectively, our findings highlight potential novel therapeutic targets for cervical carcinoma, particularly in under-vaccinated regions, and emphasize the importance of combining these strategies with vaccination efforts to reduce cervical cancer mortality across the world.

## Materials and methods

### Ethical aspects

This study was approved by the Research Ethics Committee from Clinical Hospital, Ribeirao Preto Medical School, University of Sao Paulo (3.130.450). Experiments using animal cohorts were approved by the Ethics Committee on the Use of Animals of the Ribeirao Preto Medical School (003/2015–1 and 108/2020).

### Research participants

The study's objectives were clarified in the Informed Consent Form and explained to all participants. Inclusion criteria: i) age 18–55; ii) genitoscopy with high-grade lesion in the uterine cervix; iii) biopsy confirming tumor. Exclusion criteria: i) pregnancy; ii) biopsy confirming non-malignant cells. Two biopsies were collected from each patient: one from normal tissue and one from the lesion. All samples were HPV-positive.

### Biopsy processing and tumor evaluation

The biopsies were kept at 4–8 °C overnight, followed by: (i) dehydration in alcohols of increasing concentration for 40 min each; (ii) diaphanization twice in xylol for 30 min; (iii) paraffin embedding with two liquid paraffin incubations of 1 hour 30 min each, using the Histoembedder EG1160 (LEICA, Wetzlar - Germany) [20]. HSIL grading evaluated epithelial stratification, differentiation, pleomorphism, hyperchromasia, nucleus-cytoplasm ratio, and atypical mitoses. Histological abnormalities affected at least two-thirds of the epithelium. Biopsies were blindly evaluated by Prof. Alfredo Ribeiro Silva, PhD (FMRP-USP) [21].

### Immunohistochemistry

Immunohistochemistry targeted the proteins: Matriptase (R&D, AF3946, 0.5μg/mL), KLK5 (Abcam, ab28565, 2.0μg/mL), KLK7 (R&D, AF2624, 0.125μg/mL), LEKTI (Sigma, A96960, 2.0μg/mL), HAI-1 (R&D, HID01, 0.166μg/mL), and HAI-2 (R&D, IWO01, 1.0μg/mL). Samples were deparaffinized with xylene, rehydrated through alcohol gradients, and underwent antigen retrieval via microwave. After PBS washes, methanol blocked endogenous peroxidase with 3 % H2O2. Samples were incubated with primary antibodies in 1 % BSA overnight at 4 °C. Negative controls used rabbit, goat, and mouse IgG. Secondary antibodies (Vector laboratories, California, USA) were used accordingly: BA-1000 (KLK5, LEKTI), BA-9500 (HAI-1, HAI-2), BA-9200 (KLK7), and BA-6000 (Matriptase) [14].

### Generation of KLK5/ KLK7 knockout mouse

The mouse model was developed in Dr. Thomas H. Bugge's lab (NIDCR, NIH, Bethesda, MD, USA). Two 20-nt target DNA sequences for each gene were selected using the CRISPR design website to disrupt KLK5 and KLK7 genes. The sequences generated sgRNAs that form a riboprotein complex with spCas9. Guide sequences were: KLK5: 5′-TGGCGAGGACCGGACACCCC-3′, 5′-GGCTACCCTGATCACAACCC-3′; KLK7: 5′-AAGACAGCAGCAACAGTTATC-3′, 5′-TCTTTAGCCCTGGAAACGGC-3′. The sense and antisense oligos were annealed and cloned into the px330 plasmid (Addgene, Cat. N° 42,230) using BbsI (New England Biolabs). Clones were confirmed by DNA sequencing using a U6 promoter primer. The constructs were microinjected into the male pronucleus of 2-cell embryos from FBV/NJ mice, generating 23 founders with indels. These founders were bred with wild-type FVB mice to establish colonies. Mice were housed in solid-bottom cages under controlled conditions, with experiments littermate-controlled and randomly allocated.

### Tissue thickness analysis

Ear thickness of transgenic mouse epithelia was measured with a pachymeter and analyzed using GraphPad Prism 5.0 (GraphPad Software, San Diego, CA, USA) (Supplementary Figure 1). Tissues were fixed in 4 % paraformaldehyde for 24 h, hydrated in decreasing alcohol series, stained with H&E, dehydrated in increasing alcohol series, and mounted with Fluormount. Slides were examined, and images were captured using an Olympus BX61VS microscope [22].

### Clinical score

The phenotypic analysis of the animals and the monitoring of spontaneous lesions over time are highly subjective. To reduce the bias caused by the observer's experience, we adapted a lesion quantification system used by dermatologists worldwide. Initially proposed by the European Task Force on Atopic Dermatitis in 1992, this system scores lesions based on criteria such as location, extent, severity, and behavioral symptoms to examine cases of Atopic Dermatitis [23]. It was validated and named SCORAD (Scoring for Atopic Dermatitis). In this study, we adapted the scale used in humans for application in mice. Since all lesions appeared in the head region of the animals, our quantification focused on analyzing lesion severity, grading it from 1 to 7 (**Supplementary Figure 3**). For this purpose, monthly photographs of the mice were taken, starting at the third month and continuing until the twelfth month of life.

### PAR-2 and NFκB activation assay

HEK293T cells were plated in 6-well plates and cultured in Dulbecco's Modified Eagle MediumGibco–Life Technologies) with 10 % fetal bovine serum for 24 h. Cells were co-transfected with pSRE or NFκB-firefly luciferase reporters (1000 ng, Stratagene) and pRL-Renilla luciferase (500 ng), along with pcDNA 3.1-PAR2 (1000 ng, Missouri S&T cDNA Resource Center) using Lipofectamine 3000 (Life Technologies). After 16 h, cells were transferred to 96-well plates (60,000 cells/well) and stimulated for 7 h with different kallikreins. Luciferase activity was measured with the Dual-Luciferase Assay Kit (Promega) using a Varioskan Lux (Thermo Fisher – Invitrogen), and the Firefly/Renilla luciferase ratio determined SRE activity [15].

### Bulk RNA-seq

Total RNA was extracted from newborn Klk5/Klk7^+/+^;K14-HPV^+/0^ (*n* = 3) and Klk5/Klk7^-/-^;K14-HPV^+/0^ mice (*n* = 2) using RNeasy (Qiagen, USA). RNA was quantified and analyzed via RNA-Seq following Illumina guidelines (Illumina, USA). Libraries were sequenced on the Illumina NovaSeq 6000 platform (50 bp paired-end reads) and aligned to GRCm39 (mm10) using STAR (v.2.7.10a) (Dobin et al., 2013). PCR duplicates were removed, and gene quantification was performed with HTSeq software (v.2.0.1) (Anders & Huber, 2010). Differential expression analysis was conducted using DESeq2 (v.3.15) (Mi et al., 2014). The RNAseq heatmap displayed expression profiles of eight genes (*Nelfcd, CD39, Ppp1r14b, Arghef18, Phtf12, Efl1, NFkB1, Dennd4a*) across the five samples (*Klk5/Klk7^+/+^;K14-HPV^+/0^*(*n* = 3) and *Klk5/Klk7^-/-^;K14-HPV^+/0^* mice (*n* = 2)), with hierarchical clustering grouping genes and samples with similar patterns [24].

### Real-time PCR

Total RNA was extracted from tissue samples of Klk5/Klk7^+/+^;K14-HPV^+/0^ (*n* = 4) and Klk5/Klk7^-/-^;K14-HPV^+/0^ (*n* = 5) newborn mice using trizol and chloroform. cDNA was synthesized using the High-Capacity cDNA kit (Applied Biosystems, USA). Real-time PCR was performed for *Klk14* and *Gapdh* (endogenous control) using Power SYBR Green PCR Master Mix (Applied Biosystems, USA). Fluorescence was measured on a Real-Time PCR 7500 system and analyzed with SDS v1.3 (Applied Biosystems, USA). Results were expressed as CT values normalized to the endogenous *Gapdh* gene [15].

### Rho GTPase pull-down assay

Plasmid-transfected HEK293T cells were serum-starved for 4 h. Following a 5-minute treatment with Thermolysin or Thermolysin combined with KLK14, cells were lysed on ice using a protease inhibitor-supplemented lysis buffer. Lysates were centrifuged, and supernatants were incubated with glutathione S-transferase-rhotekin-Rho-binding domain on glutathione-sepharose beads. After incubation, GTP-bound Rho was extracted, and samples were analyzed using a western blot [25].

### Immunoblot analysis

Immunoblot analysis followed cell lysis and heating samples to 95–100 °C for 5 min. Lysates were subjected to SDS-PAGE and transferred to polyvinylidene difluoride membranes (Millipore, USA). Membranes were incubated with anti-RhoA antibody (1:1000, Cat No: 2117, Cell Signaling Technology, USA) followed by goat anti-rabbit secondary antibody (1:50,000, Southern Biotech, USA). Detection was done using Immobilon Western Chemiluminescent HRP substrate (Millipore, USA) [26].

### Statistical analysis

Statistical analysis was performed using GraphPad Prism 5.0 (GraphPad Software, USA). Data distribution was assessed using the D'Agostino & Pearson and Shapiro-Wilk normality tests. Paired *t*-tests were used to compare expected and lesion biopsies (HSIL) when data followed a normal distribution; otherwise, the Wilcoxon signed-rank test was used. Results were expressed as mean ± standard deviation (SD), with p-values <0.05 considered significant. Two-way ANOVA with Bonferroni post-test was applied for mouse ear thickness, total SCORE, and qPCR. Mann-Whitney *U test* was used for PAR-2 and RhoA activation analysis. For the clinical correlation, statistical analysis was performed using Pearson's Chi-squared test with Yates' continuity correction (RStudio). Protein expression HSIL was divided into high and low according to the mean of stained area (%) for matriptase (51,49), HAI-1 (53,26), HAI-2 (37,20), KLK5 (36,14), KLK7 (24,61) and LEKTI (17,51).

## Results

### Differential expression and localization serine proteases in biopsies from patients with early cervical carcinoma (high-grade intraepithelial lesion - HSIL)

**Fig. 1:**
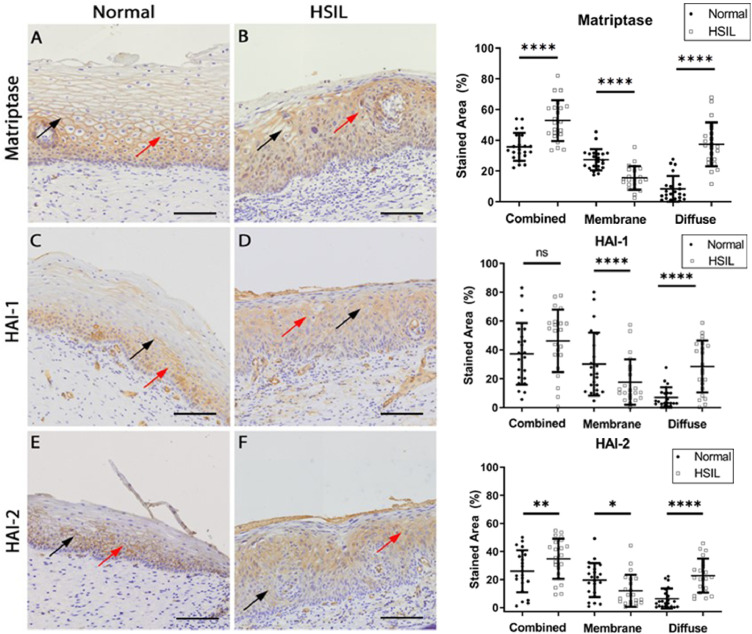
***Expression of Matriptase, HAI-1, and HAI-2 is altered in HSIL.*** (A-F) Representative images of histologically normal cervical (A, C, and E) and HSIL (B, D, and F) biopsies stained for Matriptase (*n* = 22), HAI-1 (*n* = 22) and HAI-2 (*n* = 22), respectively. Black arrows point to specific staining. (G, H, and I) Quantification of matriptase, HAI-1, and HAI-2 stainings, respectively (percentage of combined membrane and diffuse expression). Statistical analysis: paired *t*-test and Wilcoxon signed-rank test (Matriptase – combined and membrane: *p* < 0.0001, both paired *t*-test, diffuse: *p* < 0.0001, Wilcoxon signed-rank test; HAI-1 – combined: NS, paired *t*-test, membrane and diffuse: *p* < 0001, both Wilcoxon signed-rank test; HAI-2 – combined: *p* < 0.0008, paired *t*-test, and membrane diffuse::*p* < 0.001 and *p* < 0.0001, both Wilcoxon signed-rank test). Scale bar: 50 µm.

**Fig. 2:**
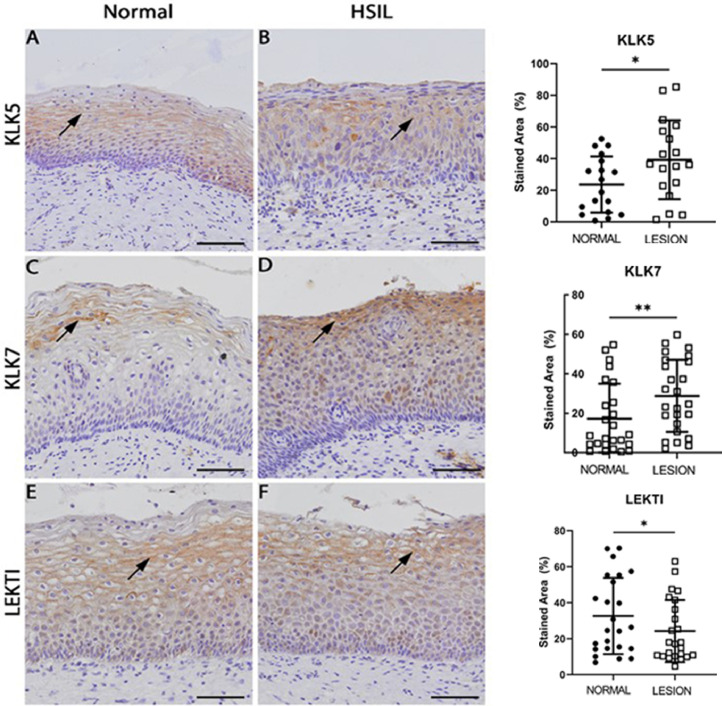
***Expression of KLK5 and KLK7 is altered in HSIL.*** (A-F) Representative images of histologically normal cervical (A, C, and E, normal, *n* = 26) and HSIL (B, D, and F, *n* = 26) biopsies stained for KLK5, KLK7, and LEKTI, respectively. Black arrows point to specific stainings. (G, H, and I) Quantification of KLK5 (*n* = 18), KLK7, and LEKTI (*n* = 26) staining, respectively (percentage of the expression of the total epithelial). Statistical analysis: paired *t*-test and Wilcoxon signed-rank test (KLK5, *p* = 0,0038, Wilcoxon test; KLK7, *p* = 0,0011, Wilcoxon test, and LEKTI, *p*=NS). Scale bar: 50 µm.

Firstly, we investigated the expression of matriptase and its respective inhibitors, HAI-1 and HAI-2, using immunohistochemistry on healthy tissue and HSIL samples. Consistent with their role as membrane-associated proteins, matriptase, HAI-1, and HAI-2 were predominantly localized at the cell membrane (Fig. 1**A–F, black arrows**)**.** Interestingly, HSIL exhibited a more diffuse expression pattern than controls (Fig. 1**A–F, red arrows**)**.** Quantitative analysis revealed that HSIL demonstrated a higher overall expression (membrane plus diffuse) of matriptase, HAI-1, and HAI-2 (Fig. 1**G–I**). However, membrane-specific expression of these proteins was more significant in normal tissues than in HSIL samples (Fig. 1**G–I**). Diffuse expression of matriptase, HAI-1, and HAI-2 significantly increased in HSIL compared to controls (Fig. 1**G–I**)**.** KLK5 and KLK7 were notably overexpressed in HSIL samples (Fig. 2)**.** Immunostaining revealed a pericellular distribution for these proteases (Fig. 2**A–D, black arrows**)**,** with significantly higher levels of KLK5 and KLK7, as well as their inhibitor LEKTI, in tumors compared to normal tissues (Fig. 2**G-I**)**.**

Table 1 displays the clinical characteristics of the included patients in our study, all of whom presented with high-grade squamous intraepithelial lesions (HSIL) and tested positive for HPV. The patient cohort was composed of women predominantly aged 36 to 45 years, with a majority identifying as white (73.08 %). Regarding marital status, most participants were either married (42.31 %) or single (23.08 %). A low prevalence of current smoking (19.23 %) and alcohol use (7.69 %) was observed in the cohort. In terms of pre-existing conditions, most participants (61.54 %) had no comorbidities, although cases of hypertension (15.38 %) and HIV infection (11.54 %) were identified. No clinical correlation was observed regarding protein expression.

**Table 1:** ***Patient demographic data.*** Demographic characteristics of study participants (*n* = 26) diagnosed with high-grade squamous intraepithelial lesion (HSIL) and positive for HPV.

	n [26]	%
Age		
30–35	1	3,85
36–40	11	42,31
41–45	7	26,92
46–50	3	11,54
51–55	2	7,69
56–60	1	3,85
61–70	1	3,85
Marital Status		
Single	6	23,08
Married	11	42,31
Cohabiting partner	5	19,23
Divorced	3	11,54
Widowed	1	3,85
*Race/ethnicity		
White	19	73,08
Black	1	3,85
*Parda*	6	23,08
Smoking status		
Present smoker	5	19,23
Non-smoker	21	80,77
Alcohol use status		
Alcohol user	2	7,69
Non alcohol user	24	92,31
Number of pregnancies		
0	8	30,77
1	2	7,69
2	7	26,92
3	3	11,54
4	3	11,54
5	1	3,85
≥6	2	7,69
Contraceptive method		
Tubal ligation	6	23,08
IUD	1	3,85
Etonogestrel implant	2	7,69
Oral contraceptive pill	6	23,08
DMPA injectable contraceptive	3	11,54
No contraceptive method	8	30,77
Pre-existing conditions		
HTN	4	15,38
HIV+	3	11,54
Hypothyroidism	1	3,85
Schizophrenia	1	3,85
Hansen’s disease	1	3,85
Without comorbidities	16	61,54

⁎ Race/ethnicity was self-reported based on Brazilian Census categories (White, Black, Pardo, Indigenous, and Asian). IUD: Intrauterine device COC: Combined oral contraceptive DMPA: Depot medroxyprogesterone acetate.

### KLK5/KLK7 double deficiency reduces HPV-dependent tumor progression

As HPV infection is a key driver for tumor development in cervical cancer, we sought whether the HPV oncogenes could impact these alterations in serine protease expression. To this end, we crossed *K14-HPV^+/0^* mice, which express the HPV oncogenes in the basal layer of the epithelia and develop spontaneous tumors derived from keratinocytes, with mice overexpressing matriptase cognate inhibitors HAI-1 (*K5-Spint1^+/0^*) or HAI-2 (*K5-Spint2^+/0^*). Interestingly, mice lacking either HAI-1 (*K5-Spint1^+/0^;K14-HPV^+/0^*) **(Supplementary Figure 1A-B)** or HAI-2 (*K5-Spint2^+/0^;K14-HPV^+/0^*) **(Supplementary Figure 1C-D)** exhibited similar clinical score compared to *K14-HPV^+/0^* transgenic mice, indicating that matriptase inhibition by either HAI-1 or HAI-2 does not modulate HPV-dependent phenotype.

**Fig. 3:**
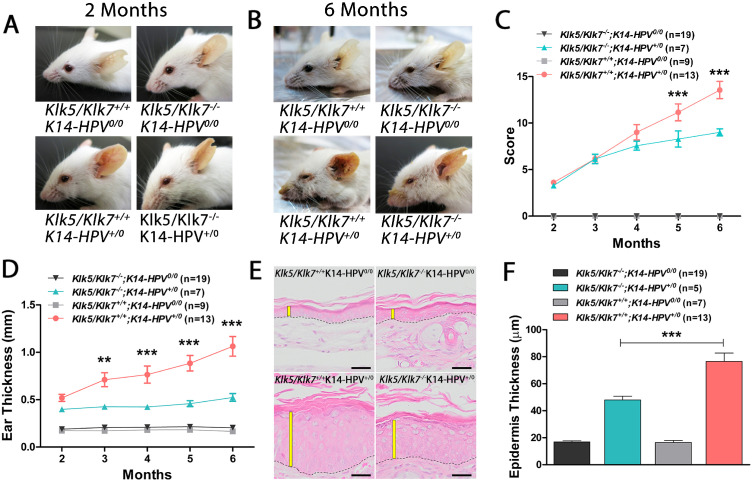
***Klk5/Klk7 double deficiency delays HPV-dependent premalignant progression****.* (A) Representative images of 2-month-old mice: Klk5/Klk7+/+;K14-HPV0/0 (upper left image), Klk5/Klk7-/-;K14-HPV0/0 (upper right image), Klk5/Klk7+/+;K14-HPV+/0 (lower left image) and Klk5/Klk7-/-;K14-HPV+/0 (lower right image). (B) Representative images of 6-month-old mice: Klk5/Klk7+/+;K14-HPV0/0 (upper left image), Klk5/Klk7-/-;K14-HPV0/0 (upper right image), Klk5/Klk7+/+;K14-HPV+/0 (lower left image) and Klk5/Klk7-/-;K14-HPV+/0 (lower right image) showing a partial rescue of the phenotype in the Klk5/Klk7-/-;K14-HPV+/0 mice when compared with Klk5/Klk7+/+;K14-HPV+/0 mice. (C) SCORE analysis quantification of mice shown in Figs. 3A and 3B (Two-way ANOVA, followed by Bonferroni post-test, *p* < 0.001). (D) Quantification of the ear thickness in mice is shown in Figs. 3A and 3B (Two-way ANOVA, followed by Bonferroni post-test, *p* < 0.001). (E) Representative H&E staining showing the thickness of Klk5/Klk7+/+;K14-HPV0/0 (upper left image), Klk5/Klk7-/-;K14-HPV0/0 (upper right image), Klk5/Klk7+/+;K14-HPV+/0 (lower left image) and Klk5/Klk7-/-;K14-HPV+/0 (lower right image) epidermis. (F) Quantification of epidermis thickness. (One-way ANOVA, followed by Bonferroni post-test, *p* < 0.05).

To address whether the double deficiency of *Klk5* and *Klk7* could modulate HPV-dependent phenotype, we generated mice genetically deficient for both *Klk5* and *Klk7*. In this way, we used CRISPR/Cas9 approaches to disrupt exon 1 of *Klk5* (**Supplementary Figure 2A**) and exon 2 of *Klk7* (**Supplementary Figure 2B**). KLK5- and KLK7-null founders were confirmed after sequencing the genomic DNA (**Supplementary Figure 2C**) and immunostaining for anti-KLK5 and anti-KLK7 (**Supplementary Figures 2D and E**). Remarkably, HPV transgenic mice lacking *KLK5* and *KLK7* (*Klk5/Klk7^-/-^K14-HPV^+/0^*) have rescued the HPV-dependent phenotype after two and six months (Fig. 3**A and B**). In addition, the clinical score for *Klk5/Klk7^-/-^K14-HPV^+/0^* was significantly lower compared to *Klk5/Klk7^+/+^K14-HPV^+/0^* mice (**Figure C**). We found that *Klk5/Klk7^-/-^K14-HPV^+/0^* mice presented lower ear thickness when compared to the *Klk5/Klk7^+/+^K14-HPV^+/0^* mice (Fig. 3**D**). Consistently, the histopathological analysis showed that epidermis from *Klk5/Klk7^+/+^K14-HPV^+/0^* mice is thicker than controls (Fig. 3**E-F**). Altogether, our data show that the double deficiency of *Klk5* and *Klk7* restores lesion progression in transgenic HPV-dependent mice.

### Double deficiency of Klk5 and Klk7 in HPV-transgenic mice modulates PAR-2 activation

**Fig. 4:**
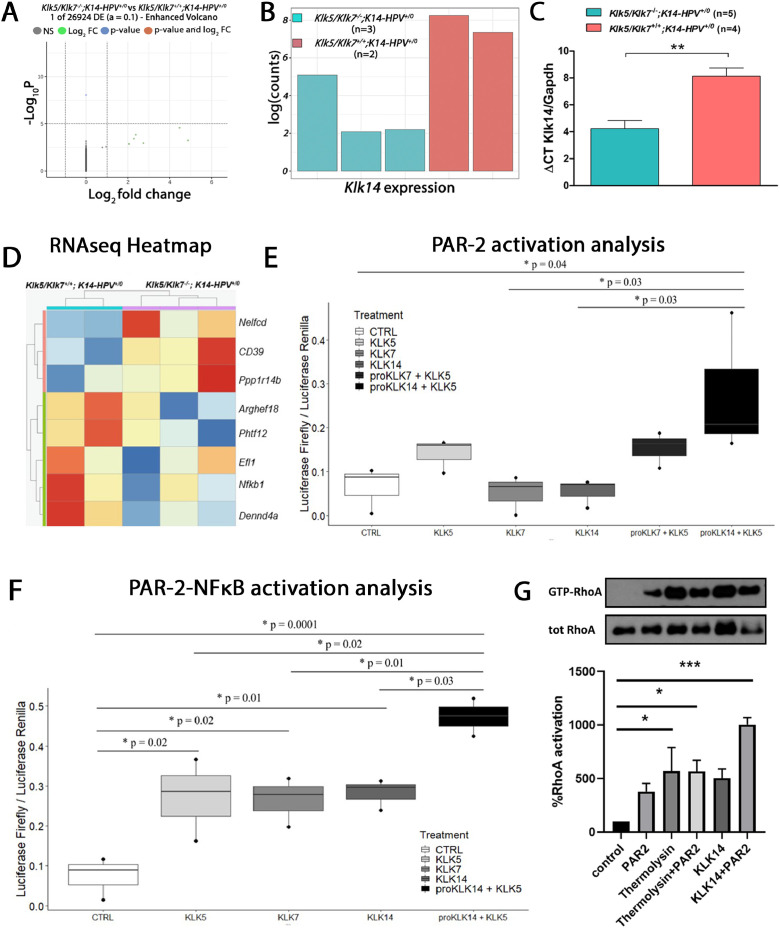
***KLK14-dependent PAR-2 activation signals through RhoA and NFκB.*** (A) Volcano plot from the RNA-seq showing a global view of the results. The Klk14 has the lowest adjusted p-value. (B) The count plot highlighted the expression levels of the Klk14, the most highly differentially expressed gene from RNA-seq. (C) qPCR of Klk14 of RNA extracted from Klk5/Klk7+/+;K14-HPV+/0 (*n* = 4) and Klk5/Klk7-/-;K14-HPV+/0 (*n* = 5) mouse epidermis, showing that Klk14 expression is higher in Klk5/Klk7+/+;K14-HPV+/0 mouse. (D) RNAseq heatmap depicting gene expression patterns in HPV transgenic mice bearing KLK5 and KLK7 versus HPV transgenic mice lacking Klk5 and Klk7. (E) KLK5-, KLK7-, KLK14, KLK7-KLK14- and KLK5-KLK14-mediated PAR-2 activation analysis. HEK293T cells were transfected with pCDNA 3.1-PAR-2, pRL-Renilla luciferase, and SRE-Firefly luciferase reporter plasmids and treated with hrKLK5, hrKLK7, hrKLK14, or the combination for six h, PAR-2 activation was measured by Luciferase activity. rhKLK5 was used as a positive control for PAR-2 activation (One-Way ANOVA with Tukey’s post-hoc test). (F) KLK5-, KLK7-, KLK14 and KLK5-KLK14-mediated PAR-2-NFκB activation analysis. HEK293T cells were transfected with pCDNA 3.1-PAR-2, pRL-Renilla luciferase, and NFκBRE-Firefly luciferase reporter plasmids and treated with hrKLK5, hrKLK7, hrKLK14, or the combination for six h, NFκB activation was measured by Luciferase activity (One-Way ANOVA with Tukey’s post-hoc test). (G) A representative Western blot shows the results of a RhoA pulldown assay in PAR-2 transfected HEK293 cells treated with rhKLK14 (activation of proKLK14 recombinant protein was performed using thermolysin). Total cell lysates (input) and RhoA-bound (active) fractions were immunoblotted with antibodies against RhoA and total RhoA (loading control). The RhoA-bound fraction reflects the activated, GTP-bound form of RhoA. (Mann-Whitmey *U* test).

To elucidate the downstream mechanisms associated with protecting HPV-mediated lesions in transgenic mice lacking *Klk5* and *Klk7*, we performed bulk RNA-seq analysis in lesion samples (Fig. 4**A**)**.** Our data revealed that *Klk14*, another serine protease, was the only significantly downregulated gene in Klk5/Klk7-/-K14-HPV^+/0^ mice compared to their Klk5/Klk7+/+K14-HPV^+/0^ littermates (Fig. 4**B**). This finding was corroborated through qPCR validation (Fig. 4**C**).

To identify molecular targets affected by *Klk14*, we further analyzed our RNA-seq data and generated a heatmap highlighting distinct gene clusters with coordinated expression patterns. Notably, genes associated with immune regulation and transcriptional process, such as *Entpd1* and *Nelfcd*, were upregulated in lesions from Klk5/Klk7-/-K14-HPV^+/0^ mice (Fig. 4**D**). Conversely, genes implicated in tumor progression, including *Arghef18* and *NfκB1*, were downregulated in HPV transgenic mice lacking *Klk5* and *Klk7*.

To explore the functional relevance of our findings, we employed a reconstituted cell-based assay using HEK293 cells transfected with a PAR-2 expression vector and a serum response element (SRE)-luciferase reporter plasmid. Cells were treated with recombinant human KLK5 (rhKLK5), KLK7 (rhKLK7), and KLK14 (rhKLK14). Notably, KLK5-mediated activation of PAR-2 promoted KLK14 activation, leading to increased transcriptional activity. In contrast, KLK7-dependent activation of PAR-2 did not activate KLK14 (Fig. 4**E**).

Given the established association between PAR-2 activation and NFκB [27] activity, we next investigated the role of KLK5 in KLK14-dependent NFκB activation (Fig. 4**F**). Reporter assays using HEK293 cells transfected with a PAR-2 expression vector and an NFκB response element (NFκBRE)-luciferase reporter plasmid demonstrated increased NFκB transcriptional activity upon KLK5-dependent KLK14 activation.

Additionally, considering that *Arghef18*, a Rho guanine nucleotide exchange factor involved in Rho GTPase regulation, was downregulated in HPV transgenic mice lacking *Klk5* and *Klk7* (Fig. 4**G**), and given the involvement of the RhoA/ROCK pathway in NFκB signaling [28], we conducted a RhoA pull-down assay. This assay evaluated the potential synergistic effect of thermolysin-activated KLK14 with PAR-2 expression on RhoA pathway activation, which may contribute to NFκB signaling activation. Interestingly, KLK14 enhanced RhoA activation in a PAR-2-dependent manner (Fig. 4**G**). Our findings demonstrate that HPV transgenic mice lacking *Klk5* and *Klk7* exhibit impaired activation of KLK14, which plays a pivotal role in regulating signaling pathways associated with carcinogenesis, such as NFκB and RhoA signaling.

## Discussion

In this study, we demonstrated that the expression of serine proteases matriptase, KLK5, KLK7, and their inhibitors, HAI-1 and HAI-2, is dysregulated in human biopsies of high-grade squamous intraepithelial lesions (HSIL). We also showed that genetic deficiency of both *Klk5* and *Klk7* rescues the HPV-dependent lesion in transgenic mice by a mechanism dependent on *Klk14* expression.

We first evaluated the epithelial expression of matriptase, HAI-1, and HAI-2 in normal and HSIL biopsies, which revealed that matriptase, HAI-1, and HAI-2 are overexpressed and exhibit diffuse distribution. These findings align with previous studies reporting aberrant matriptase expression in various cancers, including cervical carcinoma [7,15,29]. Matriptase activation in stratified epithelia relies on a reciprocal zymogen activation complex with prostasin, which is inhibited by HAI-1 and HAI-2 [16]. HAI-1 deficiency does not affect matriptase subcellular localization in the intestinal epithelium, while HAI-2 loss results in matriptase overexpression due to increased shedding [17]. Considering this, we hypothesized that overexpression of HAI-1 and HAI-2 might prevent matriptase-driven carcinogenesis through inhibitory complex formation, and HAI-2 overexpression likely disrupts matriptase subcellular localization.

Since matriptase knockout mice are non-viable [18], we employed transgenic models overexpressing HAI-1 under the keratin-5 (K5) promoter to investigate matriptase's role in HPV-dependent carcinogenesis. Notably, in a DMBA-induced carcinogenesis model, the malignant phenotype observed in K5-matriptase mice was abolished when HAI-1 was co-expressed [7]. However, our results showed that co-expression of either HAI-1 or HAI-2 with HPV oncogenes did not rescue the HPV-dependent malignant phenotype.

We further examined the expression of KLK5, KLK7, and their inhibitor LEKTI in human biopsies. LEKTI downregulation is associated with multiple cancers, including non-melanoma skin cancer, head and neck squamous cell carcinoma, and esophageal cancer [7,12,28], and its overexpression has been shown to inhibit the Wnt/β-catenin pathway, reducing tumor cell proliferation and invasion [12]. In HSIL samples, we found significant overexpression of KLK5, KLK7 and their inhibitor LEKTI, which is consistent with reports in other cancers, such as colon and oral carcinomas [8,14,28].

Activation of KLK5 induces inflammation and atopic-like lesions via the PAR-2 signaling pathway [11]. Transgenic models overexpressing *Klk5* exhibit features of atopic dermatitis, including corneodesmosome degradation, detachment of the stratum corneum, and increased cytokine production [16]. Similarly, *Klk7* overexpression results in epidermal thickening and dermal inflammation resembling atopic dermatitis [30]. Our findings confirm that both *Klk5* and *Klk7* exacerbate HPV-dependent histopathological features by modulating *Klk14* expression in cervical carcinogenesis.

KLK14-dependent activation of PAR-2, NF-kB, and RhoA signaling suggests a complex interplay between protease signaling, inflammation, and cytoskeletal dynamics in HPV-associated cancers. *Klk14* expression has also been linked to prostate and breast cancers [31,32]. Our results uniquely demonstrate that RhoA signaling is activated via KLK14-mediated PAR-2 mechanisms. RhoA activates NF-kB signaling by regulating IκB kinase (IKK), promoting phosphorylation and nuclear translocation [33]. Additionally, RhoA-mediated cytoskeletal rearrangements and integrin-dependent signaling contribute to NF-kB activation [34,35]. Targeting KLK5, KLK7, and KLK14 may pave the way for novel treatments that prevent cancer progression. While cervical cancer is largely preventable through HPV vaccination and regular screening, many low- and middle-income countries face significant challenges in implementing effective vaccination programs [36].

Taken together, the immunohistochemical expression of matriptase, HAI-1, and HAI-2, from a predominantly membranous localization to a more diffuse pattern in HSIL lesions, indicates a loss of precise spatial control over proteolytic activity, suggesting an activity modulation of these proteases and their inhibitors within the pre-malignant lesion microenvironment. This spatial alteration, together with the highly specific activation hierarchy of KLK14 by KLK5, but not by KLK7, via PAR-2, illustrates a precise mechanism within the kallikrein cascade that dictates tumor progression, indicating that different members of the same protease family exert distinct regulatory roles. Additionally, the dual deficiency of KLK5/KLK7 induces a multifaceted transcriptional reprogramming, impacting not only pro-tumorigenic signaling pathways such as RhoA/NF-κB, but also broadly modulating genes related to immune regulation and transcriptional processes. This broad alteration in the gene expression program suggests a systemic readjustment of cellular homeostasis, contributing to the rescue of the malignant phenotype and highlighting the complexity of the molecular interaction network in cervical carcinogenesis.

In conclusion, this study underscores the pivotal roles of KLK5, KLK7, and KLK14 in cervical carcinogenesis driven by HPV infection. Our findings suggest that serine protease inhibitors could serve as valuable targets to develop novel treatments for cervical cancer.

## Funding

This study was financed, in part, by the São Paulo Research Foundation (FAPESP), Brasil. Processes Numbers #2019/04896-5, 2021/14597-5 and 2024/10667-7) and 10.13039/501100008353Fundação de Apoio ao Ensino, Pesquisa e Assistência do Hospital das Clinicas da Faculdade de Medicina de Ribeirão Preto da Universidade de São Paulo (FAEPA) research grants to K.U.S. G.V.V.-Ph.D and R.A.A.d.S.-MSc fellowship from Brazilian Federal Agency for Support and Evaluation of Graduate Education (CAPES, an agency linked to the 10.13039/501100006366Ministry of Education of Brazil). B.B.M.G.-Msc fellowship from FAPESP #2016/16715-7. E.Z.M.S- post-doctoral fellowship FAPESP #2016/13228-8 and FAPESP BEPE fellowship #2017/24730-9. B.M.I.B.- fellowship from FAPESP #2022/01968-8. V.M.A.- fellowship from FAPESP # 2019/25900-0. K.L.L-D. - Msc fellowship from FAPESP # 2024/13367-4. The funders had no role in study design, data collection, analysis, publication decision, or manuscript preparation.

## Ethics statement

The study was conducted according to the guidelines of the Declaration of Helsinki and approved by the Ethics Committee on Human Research of Ribeirão Preto Clinical Hospital and Ribeirão Preto Medical School, University of São Paulo (protocol #3.130.450). All experiments involving mice were approved by the Ethics Committee on Animal Research of Ribeirao Preto Medical School, University of São Paulo 2015) and are by the Guidelines of the Brazilian College of Animal Experimentation.

## Informed consent statement

The objectives of this study were clarified in the Informed Consent Form, which is available in supplementary material, and explained to all participants. Two biopsies, one from normal tissue and the other from a cervical lesion, were collected from the patients for analysis.

## Data access statement

The data supporting this study's findings are available in the published article or can be obtained from the corresponding author upon request.

## Glossary

1. **ArhGEF18 -** Required to maintain apico-basal polarity, localization of tight junctions, and cortical actin, thus shaping cellular morphology. Loss of ArhGEF18 activity results in increased proliferation and reduced cell cycle exit.
2. **Basal layer of the epithelium -** The innermost layer of the epithelium, made up of basal cells and a basement membrane. The basal layer is also known as the stratum basale.
3. **CRISPR/Cas9 -** A genetic engineering technique in molecular biology by which the genomes of living organisms can be modified. It is based on a simplified version of the bacterial antiviral defense system CRISPR-Cas9. By introducing the Cas9 nuclease into a cell, in complex with a synthetic guide RNA (gRNA), the cell's genome can be cut at the desired location, allowing existing genes to be removed and/or new ones to be added in vivo.
4. **DMBA -** 7,12-Dimethylbenz[a]anthracene, a chemical carcinogen used to induce tumors in experimental models.
5. **ENTPD1 -** The ENTPD1 gene, or ectonucleoside triphosphate diphosphohydrolase 1, codes for a protein that regulates nucleotide levels. It is also known as CD39 (Cluster of Differentiation 39).
6. **FBV/NJ mice -** An inbred strain of mouse model most commonly used for transgenic applications.
7. **GTPase -** An enzyme that transforms guanosine triphosphate (GTP) into guanosine diphosphate (GDP). It is essential for regulating various cellular processes, such as growth, differentiation, migration, and vesicle trafficking.
8. **HAI-1 -** Hepatocyte activator inhibitor type 1, a protein that regulates the activity of serine proteases, including matriptase. It is expressed on the surface of epithelial cells and placental cytotrophoblasts.
9. **HAI-2 -** Hepatocyte activator inhibitor type 2, which also regulates serine proteases and may influence tumor progression. It is found in epithelial tissues of humans and mice, but HAI-2 immunoreactivity is exclusively observed in the cytoplasm.
10. **Heatmap -** A graphical representation of data that uses a system of color coding to represent different values. Heatmaps are used in various forms of analytics but are most commonly applied to show user behavior on specific web pages or webpage templates.
11. **HEK 293T cells -** A derivative of the human embryonic kidney 293 (HEK293) cell line, modified to express the SV40 large T antigen.
12. **HSIL -** High-grade squamous intraepithelial lesions, associated with an increased risk of progression to cervical cancer.
13. **Human Papillomavirus (HPV) -** One of the most common sexually transmitted infections worldwide. It involves diverse groups of DNA viruses belonging to the Papillomaviridae family, which replicate specifically in the nucleus of squamous epithelial cells.
14. **IKK -** IκB kinase, an enzymatic complex that regulates the activation of the NF-κB pathway, promoting inflammatory and oncogenic responses.
15. **Kallikreins (KLKs) -** A family of 15 serine proteases found in the human body, involved in the regulation of blood pressure, electrolyte balance, and tissue repair. These kallikreins release kinins by acting on kininogens.
16. **LEKTI -** A serine protease inhibitor derived from epithelial lymphopoiesis, involved in skin homeostasis and inflammation regulation.
17. **Luciferase -** An enzyme that catalyzes the chemical reaction between luciferin and oxygen, producing light. It is found in fireflies and is used in molecular and cellular biology.
18. **Matriptase -** A transmembrane serine protease involved in cancer progression through the activation of pro-tumorigenic pathways.
19. **Nelfcd -** Neuroligin 1 (NL1) promotes the formation of glutamatergic synapses and mediates long-term potentiation. Neuroligin-1 and Glu may represent new markers of ganglion cells, whose expression may correlate with pathogenesis, diagnosis, differential diagnosis, or classification of Hirschsprung's disease.
20. **NFκB -** A nuclear transcription factor that regulates the inflammatory response and is associated with cancer progression.
21. **PAR-2 -** Protease-activated receptor 2, involved in inflammatory signaling and cancer progression.
22. **Prostasin -** A serine protease that regulates epithelial function and may modulate cancer progression.
23. **qPCR -** Quantitative PCR or real-time PCR is a variant of the polymerase chain reaction (PCR) used to amplify and simultaneously quantify the product of deoxyribonucleic acid (DNA) amplification.
24. **Reporter plasmid -** A plasmid that contains a reporter gene, which is used to detect and measure gene expression. Reporter plasmids are used to study gene expression, identify promoter and enhancer elements, and analyze protein production.
25. **RhoA -** A protein from the Rho GTPase family involved in cytoskeletal regulation, cell migration, and pro-oncogenic signaling.
26. **RNAseq heatmap -** A graphical representation of gene expression data derived from RNA sequencing (RNA-seq) experiments.
27. **Serine protease -** An enzyme that cleaves proteins by hydrolyzing peptide bonds, playing roles in digestion, coagulation, and tumor progression.
28. **SRE -** Serum Response Element, a specific DNA sequence found in the promoter region of certain genes that regulates transcription in response to extracellular signals, such as growth factors and serum stimulation.
29. **Wnt/β-catenin -** A crucial signaling pathway for cell development that is frequently deregulated in cancer.
30. **Zymogen -** An inactive enzyme precursor that requires modification, such as proteolytic cleavage, to become functional.

## CRediT authorship contribution statement

**Gabriel Viliod Vieira:** Writing – review & editing, Writing – original draft, Methodology, Investigation, Formal analysis, Data curation. **Rodrigo Alberto Alves da Silva:** Writing – review & editing, Writing – original draft, Methodology, Investigation, Conceptualization. **Letícia Andrade Costa:** Writing – review & editing, Formal analysis. **João Paulo Bianchi Ximenez:** Methodology, Investigation, Formal analysis. **Margarita Lamprou:** Methodology, Formal analysis. **Mateus Gonçalves Miranda:** Writing – review & editing, Formal analysis. **Vitor de Moura Arrais:** Methodology, Investigation, Formal analysis. **Bruna Miyoko Ikenaga de Brito:** Methodology, Investigation, Formal analysis. **Elaine Zayas Marcelino da Silva:** Methodology, Investigation, Formal analysis. **Bruno Belmonte Martinelli Gomes:** Methodology, Investigation, Formal analysis. **Carol Kobori da Fonseca:** Project administration, Methodology, Investigation, Formal analysis. **Márcia Gaião Alves:** Methodology, Investigation, Formal analysis. **Camila Aparecida Coelho Brazão:** Methodology, Investigation, Formal analysis. **Kevin Luiz Lopes-Delphino:** Writing – review & editing, Investigation, Formal analysis. **Laura Miguel Rodríguez:** Writing – review & editing, Investigation, Formal analysis. **Thiago Mattar Cunha:** Writing – review & editing, Resources, Formal analysis. **Ana Paula Lepique:** Writing – review & editing, Resources, Formal analysis. **Constantinos M. Mikelis:** Methodology, Investigation, Formal analysis. **Raphael Sanches Peres:** Methodology, Investigation, Formal analysis. **Wilson Araújo Silva:** Resources, Methodology, Investigation, Formal analysis. **Leandro Machado Colli:** Writing – review & editing, Resources, Formal analysis. **Silvana Maria Quintana:** Writing – review & editing, Resources, Formal analysis. **Katiuchia Uzzun Sales:** Writing – review & editing, Supervision, Investigation, Funding acquisition, Formal analysis, Conceptualization.

## Declaration of competing interest

The authors declare no conflict of interest.

## Appendix Supplementary materials

Image, application 1
