# Supplementary material for: KLK5 and KLK7 drive cervical carcinoma via KLK14-dependent RhoA and NF-κB pathways
Source: Transl Oncol. 2025 Aug 5;60:102488. doi: 10.1016/j.tranon.2025.102488 (PMC12375203; doi:10.1016/j.tranon.2025.102488)
Supplement: Supplementary file 1 [file mmc1.docx]

***
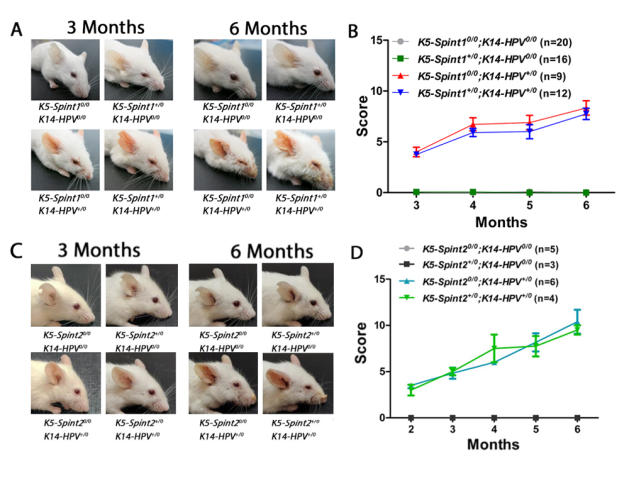
***

***Supplementary Figure 1. HAI-1 overexpression does not rescue HPV-dependent phenotype***. (A) Representative images of 3-month-old and 6-month-old mice: K5-Spint10/0;K14-HPV0/0, K5-Spint1+/0;K14-HPV0/0, K5-Spint10/0;K-14HPV+/0 and K5-Spint1+/0;K14-HPV+/0 mice. (B) Clinical score quantification of the animals mentioned in B showing that the lesions in K5-Spint10/0;K14-HPV+/0 mice are as severe as in K5-Spint1+/0;K14-HPV+/0 mice. (C) Representative images of 3-month-old and 6-month-old mice: K5-Spint20/0;K14-HPV0/0, K5-Spint2+/0;K14-HPV0/0, K5-Spint20/0;K14-HPV+/0 and K5-Spint2+/0;K14-HPV+/0 mice. (D) Clinical score quantification of the animals mentioned in B showing that the lesions in K5-Spint20/0;K14-HPV+/0 mice are as severe as in K5-Spint2+/0;K14-HPV+/0 mice. For the analysis of mouse ear thickness, total SCORE and qPCR, we used Two-way ANOVA, followed by Bonferroni post-test.

***
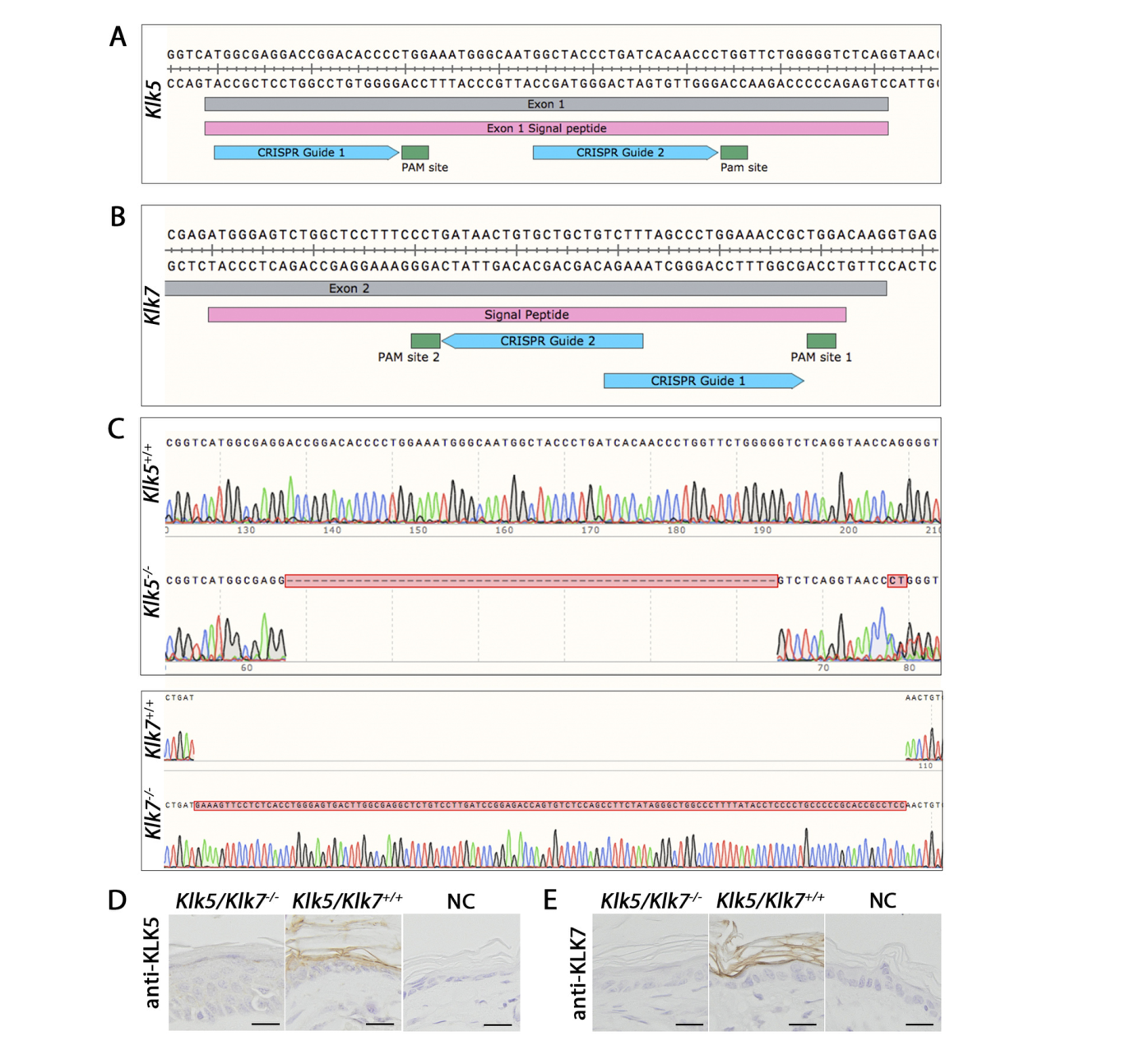
***

***Supplementary Figure 2. CRISPR-Cas9 design for generating KLK5 and KLK7 double knockout mice*.** (A and B) RNA guides (light blue arrows, CRISPR guides 1 and 2) were used to generate the knockout mice for KLK5 (targeting exon 1) (A) and for KLK7 (targeting exon 2) (B), with both exons encoding the signal peptide, using CRISPR/Cas9 technology. (C) Sequencing of the founder mice showing the disruption of the KLK5 (upper image) and KLK7 genes (lower image). (D) Immunohistochemistry of *Klk5/Klk7^-/-^* mice (left image) and *Klk5/Klk7^+/+^* (middle image) showing that KLK5 is not expressed in the *Klk5/Klk7^-/-^* mouse epithelia. (E) Immunohistochemistry of *Klk5/Klk7^-/-^* (left image) and *Klk5/Klk7^+/+^* mice (middle image) showing that Klk7 is not expressed in the *Klk5/Klk7^-/-^* mouse epithelia. Scale bars: 20 µm.

***

***

***Supplementary Figure 3. Lesion scoring from 0 to 7, being 0 no lesion and 7 severe lesions in the ear, eye and snout.*** Adaptation of the SCORAD scale for lesion quantification in mice. Lesions were classified on a scale from 1 to 7 based on severity, considering criteria such as extent and behavioral symptoms. This approach aims to reduce subjectivity in phenotypic analysis and the monitoring of spontaneous lesions.
